# En bloc resection and bone graft: does it alter the natural history of monostotic expansile fibrous dysplasia in children?

**DOI:** 10.1186/1477-7819-12-349

**Published:** 2014-11-18

**Authors:** Lianyong Li, Xiangyu Hou, Qiwei Li, Lijun Zhang

**Affiliations:** Department of Pediatric Orthopedics, Shengjing Hospital of China Medical University, Shenyang City, Liaoning Province P.R. China; Department of Pediatric Surgery, Shengjing Hospital of China Medical University, Shenyang City, Liaoning Province P.R. China

**Keywords:** Fibrous dysplasia, En bloc resection, Natural history, Skeletal immaturity

## Abstract

The surgical treatment of fibrous dysplasia remains a challenge for the pediatric orthopedist because of its high recurrence rate. Although a few successful treatments have been reported by using en bloc resection and bone graft in adults, this has not been reproduced in children. In this report, the authors present two children (2.5 and 6 years old) with monostotic expansile fibrous dysplasia involving the ulna and fibula, respectively, who underwent en bloc resection and autograft to replace the involved bones. Good bone union and functional recovery were obtained postoperatively. However, during a follow-up period of 8 and 5 years, respectively, the lesions recurred completely, and the deformities remained progressing over time. En bloc resection and bone graft cannot prevent recurrence in skeletally immature patients with monostotic expansile fibrous dysplasia, and cannot alter for the natural history of the disease. A combination of other management should be considered in children with fibrous dysplasia.

## Background

Fibrous dysplasia is an uncommon benign disorder of the bone, characterized by the progressive replacement of normal bone by fibrous tissue and dysplastic woven bone. Approximately 71.9% to 86% of fibrous dysplasia is associated with activating mutations of the *GNAS* gene [1, 2], which codes for the alpha subunit of the signaling G protein (G_s_α). Although a genetic disease, fibrous dysplasia is not inherited as the mutation occurs in the postzygotic somatocyte [3]. The range of involvement may vary from one bone (monostotic) to multiple bones (polyostotic), and it may present in association with café-au-lait skin pigmentation and endocrine disorders (McCune-Albright syndrome) [4, 5] or intramuscular myxoma (Mazabraud’s syndrome) [6]. Fibrous dysplasia represents approximately 5 to 7% of benign bone tumors [7]. The monostotic form is more frequent than the polyostotic form [8]. The common sites of skeletal involvement include the long bones, ribs, craniofacial bones, and center axial bones. The natural history of fibrous dysplasia in the limbs varies depending on the age of the patient and the form in which the lesion presents. Generally, the polyostotic lesions tend to progress more than the monostotic form, even after skeletal maturity [9]. For skeletally immature patients, the lesions may continue to enlarge over time, resulting in progressive deformities, pathological fracture, bone pain and limb-length discrepancy [10].

The treatment option for fibrous dysplasia in the limbs depends on the clinical presentation of the lesion. Surgical intervention is not necessary for an asymptomatic lesion, and clinical observation and follow-up is warranted to verify whether there has been progression. Surgical procedures may be required for correction of a limb deformity, treatment of pathological fracture, and eradication of symptomatic lesions [11, 12]. Curettage and bone graft have been used to reconstruct the involved bone in past years, but this was associated with a high rate of resorption of graft material [13, 14]. Recurrence still remains a challenge for the orthopedist. Although successful treatments have been reported in skeletally mature cases using en bloc resection and bone graft [15–17], that has not been reproduced in the skeletally immature patient.

In this report, we present two children with monostotic expansile fibrous dysplasia involving the ulna and fibula, respectively, where en bloc resection and autograft were performed to replace the involved bone. However, over a long-term follow-up the result was contrary to the previous reports. The natural history of the disease could not be reversed by surgical intervention, while a complete recurrence of the lesion and progressive deformity were observed in the affected bone.

## Case presentation

### Case 1

A 2.5-year-old boy presented to the authors’ clinic with his parents in July 2005, with a 3-month history of a firm lump and bowing deformity on his right forearm. The parents reported that the appearance of the boy’s right forearm was asymmetrical compared with the left one, and the deformity had gradually progressed during the past 3 months. No notable pain was noted. The boy had a normal physical development and denied any traumatic and medical history. The physical examination revealed the middle forearm was tumid and bowing on the ulnar and dorsal side. The patient had full range of motion on his shoulders, elbows, wrists and fingers except for a limited rotation motion on the right forearm which was 80 degrees of supination and 45 degrees of pronation. There was no tenderness to palpation over the right ulna, and the neurovascular examination was normal. The child appeared in good health on other systems.

**Figure 1:**
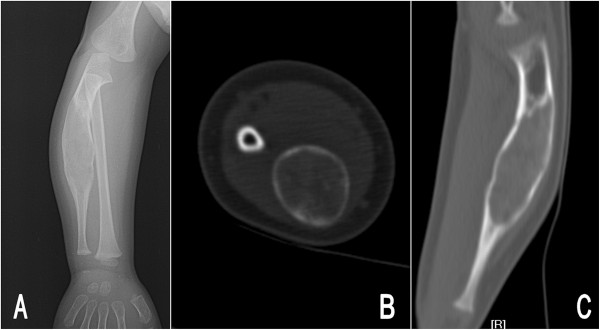
**Preoperative images of the right forearm in patient 1.** Posteroanterior radiograph **(A)** showed a fusiform-shaped, expansile ground-glass lesion in the ulna. Axial plane **(B)** and coronal reconstructed computed tomography **(C)** revealed the cortex was thinning and the area of lesion was homogeneous, with an average computed tomography value of 123 Hu.

Radiographs of his right forearm showed a large expansile lucent lesion in the diaphysis of the ulna, extending to the proximal and distal metaphysis (Figure 1A). The lesion presented as fusiform-shaped and typical “ground-glass” appearance with a size of 5.7 cm in length and 1.9 cm in maximal width. The computed tomography (CT) scan indicated it had a well defined circumscription and no calcification and bone septums were seen. The cortex showed marked expansion and thinning without periosteal reaction. The density in the area of lesion was homogeneous and the CT value was an average 123 Hu, which was similar to the density of the cancellous bone, but without the visible trabecula of bone (Figure 1B,C). No distinct soft tissue mass was exhibited on the radiographic or CT images. The laboratory tests, including complete blood cell count, thyroid function, liver and renal functions, were within normal limits.

**Figure 2:**
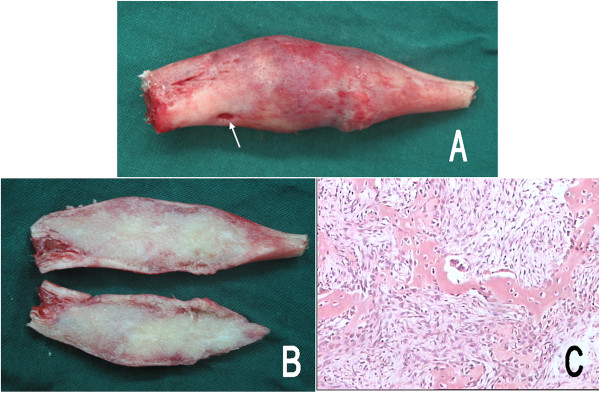
**Pathologic photographs of patient 1.** The gross specimen **(A)** appeared cortical surface expansile and rough with a small perforation (arrow), and the medullary cavity of the ulna was replaced by the solid grey white mass **(B)**. Microscopy **(C)** demonstrated “Chinese characters” woven bone was separated by abundant fibrous stroma. No osteoblast and cellular atypia were identified (hematoxylin and eosin, 100×).

Based on the clinical and typical image presentations, a diagnosis of monostotic fibrous dysplasia was established without performing an incisional biopsy. In view of the deformity progressing and limited rotation motion on the right forearm, an en bloc resection of the lesion and autogenous fibular graft was carried out. At surgery, the right ulna was exposed subperiosteally, and the lesion was excised en bloc with preservation of the proximal and distal healthy metaphysis. Then the bone defect was reconstructed using the free autogenous fibula of the ipsilateral lower leg and the intramedullary fixation was achieved with a 2.0 mm Kirschner wire. A postoperative cast was employed to immobilize the elbow and forearm in a neutral position. The excised gross sample revealed the cortical bone was thin and firm except for a small perforation on the upper pole. The medullary cavity of the ulna was replaced by a solid grey white expansile mass. The histological examination confirmed the diagnosis of fibrous dysplasia (Figure 2).

**Figure 3:**
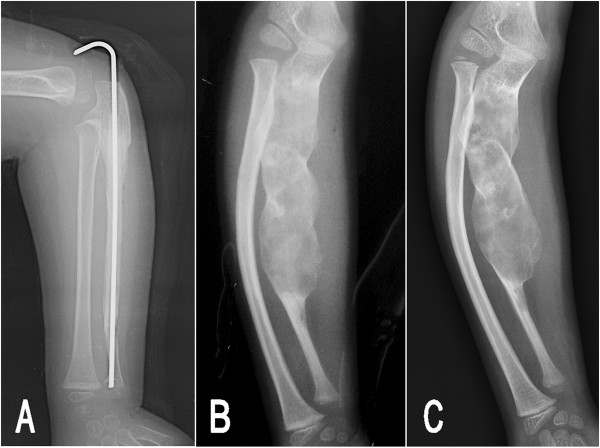
**Postoperative radiographs of patient 1.** At 6 weeks postoperation, the lateral radiograph of the right forearm **(A**) showed a well grafted union. At 4 years of follow-up **(B)**, the lesion had recurred to an enlarged extent with bowing deformity in both the ulna and radius, and the lesion and deformity continued to be progressive over time **(C)** at 8 years postoperation.

Six weeks after surgery, the patient had a well grafted union and the pin and cast were removed (Figure 3A). No early complications were seen. At 3-month follow up, he had regained the full range of rotation movement on his right forearm. However, 4 years postoperatively, the patient returned to the authors’ clinic for a reappeared deformity on his right forearm. A subsequent radiograph was taken and demonstrated a long, expansile lytic lesion in the middle and proximal ulna surrounded by an irregular thin bone shell, indicating recurrence of the tumor (Figure 3B). The lesion was enlarged and extended proximally to the coronal process level. The bowing deformity in the ulna, as well as in the radius was more severe than the initial presentations. On being informed of the natural history of fibrous dysplasia, the parents refused further treatment and determined to observe. At the last follow-up, 8 years postoperatively, the lesion and the deformity in the ulna had been further progressive, but there was no evidence of malignant transformation (Figure 3C).

### Case 2

**Figure 4:**
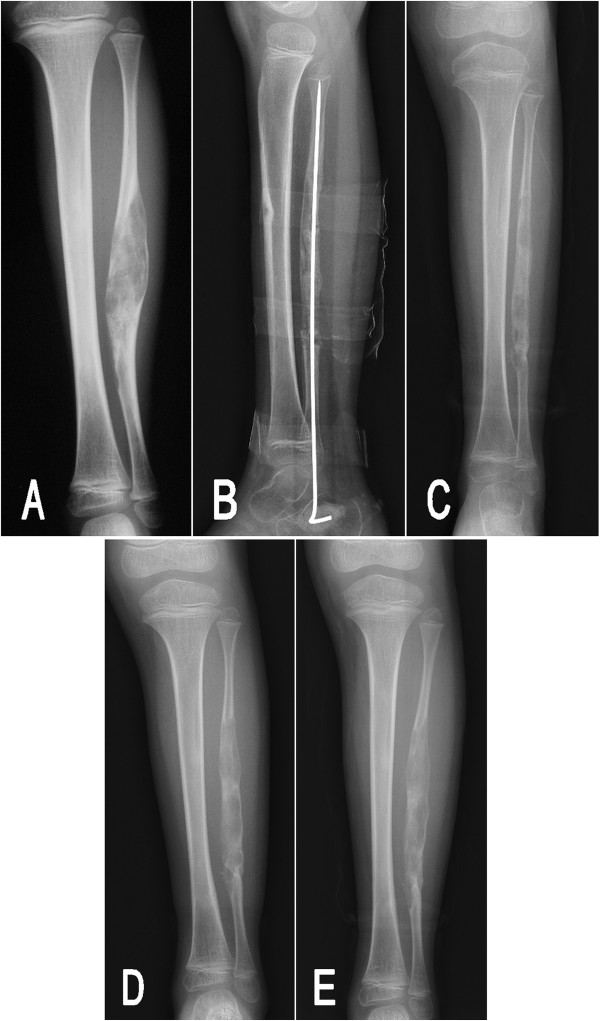
**Preoperative and postoperative radiographs of patient 2. (A)** A preoperative radiograph indicated an expansile, multiseptated lucent lesion in the fibula. **(B)** A good union and alignment had been obtained at 8 weeks postoperation. **(C)** At 4.5 months follow-up after surgery, the lesion recurred with multiple small irregular lucencies. At postoperative 2 years **(D)** and 4.5 years **(E)** of follow-up, these small lucencies gradually fused into a long, expansile lesion and appeared progressive over time.

A 6-year-old boy reported increased left lower leg pain for 1 month before presenting to the authors’ hospital. The mother observed that his pain was relieved by rest and was aggravated by activity, especially running and playing football. The pain had gradually worsened in the recent week, and there was no pain elsewhere. No analgetica or anti-inflammatory medication was used. The patient had a normal developmental history and denied any family or systematic disease history. On physical examination, the boy appeared in good health. There was mild swelling on the lateral side of the left lower leg. The patient reported a moderate tenderness when palpated over the left fibula, with no radiation of the pain. He had full range of motion on the ankles, knees and hip joints. The gait was normal and no neurovascular abnormality was noted. The posteroanterior radiograph of the left lower leg revealed a 9.0 cm long, expansile lesion in the middle fibula with resembling multiseptated, ground glass lucencies (Figure 4A). Both sides of the cortex were thinning but there was no periosteal proliferation. The radiological features showed a benign lesion of the fibula.

Subsequently, the boy underwent an en bloc resection of the fibular lesion and reconstruction with autogenous iliac bone graft. An intramedullary fixation with a 2.0 mm Kirschner wire was used to maintain the alignment of the reconstructed fibula. The postoperative pathological examination showed typical histological findings of fibrous dysplasia. Eight weeks after surgery, the grafted bone had united with the preserved proximal and distal metaphysis, and the Kirschner wire was removed (Figure 4B). At postoperative 4.5-month follow-up, the radiographs of the left lower leg demonstrated there were multiple small irregular lucencies in the fibula (Figure 4C). The extent of the lucencies was similar to the original lesion. Furthermore, in the subsequent 4.5 years of follow-up, these small cystic components had gradually fused into a long, expansile lesion and appeared progressive over time (Figure 4D,E).

## Discussion

Based on the knowledge of molecular biology, the etiology of fibrous dysplasia has been linked with an active mutation in the *GNAS* gene that occurs in somatic cells after fertilization, leading to activation of adenylate cyclase and excessive cell proliferation, but failure in differentiation to osteocyte lineage. The overproduction of disorganized fibrotic bone matrix results in the development of fibrous dysplasia [18]. All cells that derive from the mutant cells manifest the dysplastic features, and that supports the suggestion that fibrous dysplasia is caused by a single mutated cell and is a clonally derived neoplasm. Also, the transgenic experimental study by Bianco and colleagues [19] illustrated the importance of mutant cells in the pathogenesis of fibrous dysplasia. Therefore, as long as the mutant cell exists in the bone tissue, the tumorous cell proliferation is inevitable.

Due to the biological features of fibrous dysplasia, a high recurrence rate associated with curettage is possible, regardless of with or without bone grafting, because not all of the cells containing the mutation of the *GNAS* gene can be removed. For this reason, surgical removal of all dysplastic tissue comprising the mutant cells should be available for preventing recurrence. Thus, wide excision of the involved bone has been advocated in selective cases, and a few successful treatments in adult (summarized in Table 1) have been reported in the past years, by using en bloc resection of the lesion with or without bone graft [15–17, 20–22]. However, this valuable experience has not been confirmed in pediatric cases.

**Table 1:** **Previous reported treatment of fibrous dysplasia by en bloc resection**

References	Number of cases	Sex	Age (years)	Sites of lesion	Procedure	Recurrence
Verma and Paul [15]	1	F	39	Metacarpal	En bloc resection, autograft	No
Gebert *et al*. [16]	1	F	17	Radius	En bloc resection, autograft	No
Koskinen [17]	12	N/A	Adult	Limb long bones	En bloc resection, allograft	No
Traibi *et al*. [20]	7	6 M, 1 F	17-40	Ribs	Rib resection	No
Furukawa *et al*. [21]	1	F	27	Rib	Rib resection	No
Ayadi-Kaddour *et al*. [22]	10	5 M, 5 F	27-52	Rib	Rib resection	No
Current report	2	M	2.5, 6	Ulna, fibula	En bloc resection and autograft	Yes

F, female; M, male; N/A, the data was not shown in the reference.

Generally, the radiographic features of fibrous dysplasia reflect its maturation [11]. With aging, the lesion tends to become sclerotic at the edges of the lesion and shows increases in the thickness of the reactive bone. The lesion characteristically is bounded inside the sclerotic rim and appears 'stable' with no increase in the extent of lesion. On the contrary, an aggressive, unstable lesion of fibrous dysplasia is characterized by consistent replacement of the circumjacent normal cancellous and cortical bone, making the cortex thin and expansile without a distinct sclerotic rim. Andrisano and colleagues [14] divided fibrous dysplasia in children into the 'circumscribed' type and the 'extended' type according to the radiographic features. They defined a circumscribed fibrous dysplasia as any lesion that includes less than one quarter of the bone segment with only one cortex involved, and an extended type as any lesion involving more of the bone segment and both sides of diaphyseal cortex. Simultaneously, they reported the disappointing surgical result of emptying and curettage for extended fibrous dysplasia, with a failure rate of 100% in 45 cases, indicating that the extended type of lesion has a strong characteristic of activation and recurrence. The radiographic findings in the two cases presented in this report appeared aggressive, unstable and consistent with the extended type, which is obviously associated with the result of recurrence. Additionally, the age of onset is also an important cause for recurrence. Compared with the previous successful reports, the two cases in this report were very young, with an age of 2.5 and 6 years, respectively, which represents the highest probability of progression even in monostotic fibrous dysplasia. Therefore, the young age of onset and active radiographic features contributed to the failure of surgical treatment in the current two cases.

Indeed, the high probability of recurrence had been adequately considered preoperatively, and thus the procedure of en bloc resection was selected with a high expectation that it could prevent recurrence. However, unexpectedly, this has not brought any alteration in the natural history of fibrous dysplasia in the two cases. In addition to the young age and biological characteristic of the lesion itself, preservation of the periosteum around the involved bone may be also an important cause that led to recurrence. The preserved periosteum is the exclusive source of osteogenesis after en bloc resection and bone graft. We presume that the periosteum contained the cells with the mutated *GNAS* gene, and thus the production derived from the proliferation of the mutated cells was composed of fibrous tissue and immature woven bone. As internal repair and remodeling began, the normal grafted bone was replaced gradually by the dysplastic bone, and the lesion eventually reverted to its preoperative status. Maybe a radical resection involving bone lesion and corresponding periosteum could be a good option to avoid recurrence, but this could lead to a high risk of non-union. Theoretically, it is feasible to remove all pathologic periosteal tissue and preserve healthy tissue by intraoperative molecular biologic detection; however, this is technically difficult to realize.

## Conclusions

En bloc resection and bone graft have been successful for adult patient with monostotic fibrous dysplasia, but this cannot prevent recurrence in skeletally immature patients, and does not offer any alteration for the natural history of the disease. Currently, a combination of surgical intervention and pharmacologic therapy may be an optimal approach to obtain a better functional outcome [23, 24]. Although this is a negative result, we believe our unsuccessful experience has an educational value for the surgeon to understand the natural history of fibrous dysplasia and select an appropriate treatment, especially in skeletally immature patients.

## Consent

Written informed consent was obtained from the parents of the patients for publication of this case report and any accompanying images. A copy of the written consent is available for review by the Editor-in-Chief of this journal.

## Abbreviations

CT: computed tomography.
